# Supplementary material for: Resolving the full spectrum of human genome variation using Linked-Reads
Source: Genome Res. 2019 Apr;29(4):635–45. doi: 10.1101/gr.234443.118 (PMC6442396; doi:10.1101/gr.234443.118)
Supplement: Supplemental Material [file supp_gr.234443.118_Supplemental_longranger-2.2.zip › longranger-2.2.tar.bz2!/lr2.2-public/lib/stan/cmdstan/stan/doc/api/html/prod_8hpp_source.html]

Stan: src/stan/math/matrix/prod.hpp Source File


|  |  |
| --- | --- |
|  | Stan  2.5.0  probability, sampling & optimization |


- Main Page
- Related Pages
- Namespaces
- Classes
- Files

- File List
- File Members

All Classes Namespaces Files Functions Variables Typedefs Enumerations Enumerator Friends Macros Pages

- src
- stan
- math
- matrix

prod.hpp

Go to the documentation of this file.

1 #ifndef STAN\_\_MATH\_\_MATRIX\_\_PROD\_HPP

2 #define STAN\_\_MATH\_\_MATRIX\_\_PROD\_HPP

3

4 #include <vector>

5 #include <stan/math/matrix/Eigen.hpp>

6

7 namespace stan {

8  namespace math {

9

16  template <typename T>

17  inline T prod(const std::vector<T>& v) {

18  if (v.size() == 0) return 1;

19  T product = v[0];

20  for (size\_t i = 1; i < v.size(); ++i)

21  product \*= v[i];

22  return product;

23  }

24

31  template <typename T, int R, int C>

32  inline T prod(const Eigen::Matrix<T,R,C>& v) {

33  if (v.size() == 0) return 1.0;

34  return v.prod();

35  }

36

37  }

38 }

39 #endif

stan::math::prod

T prod(const std::vector< T > &v)

Returns the product of the coefficients of the specified standard vector.

**Definition:** prod.hpp:17

Eigen.hpp


---

[ Stan Home Page ]

*© 2011–2014,
Stan Development Team.*
